# Structure and co-occurrence patterns in microbial communities under acute environmental stress reveal ecological factors fostering resilience

**DOI:** 10.1038/s41598-018-23931-0

**Published:** 2018-04-12

**Authors:** Dinka Mandakovic, Claudia Rojas, Jonathan Maldonado, Mauricio Latorre, Dante Travisany, Erwan Delage, Audrey Bihouée, Géraldine Jean, Francisca P. Díaz, Beatriz Fernández-Gómez, Pablo Cabrera, Alexis Gaete, Claudio Latorre, Rodrigo A. Gutiérrez, Alejandro Maass, Verónica Cambiazo, Sergio A. Navarrete, Damien Eveillard, Mauricio González

**Affiliations:** 1 Bioinformática y Expresión Génica, Instituto de Nutrición y Tecnología de los Alimentos, Universidad de Chile, Santiago, Chile; 2 Center for Genome Regulation (Fondap 15090007), Universidad de Chile, Santiago, Chile; 3 Instituto de Ciencias Agronómicas, Universidad de O’Higgins, Rancagua, Chile; 4 Mathomics, Center for Mathematical Modeling, Universidad de Chile, Santiago, Chile; 5 LS2N, UMR CNRS 6004, IMT Atlantique, ECN, Université de Nantes, Nantes, France; 6 l’institut du thorax, INSERM, CNRS, Université de Nantes, Nantes, France; 7 Departamento de Ecología, Pontificia Universidad Católica de Chile, Santiago, Chile; 8 Institute of Ecology and Biodiversity (IEB), Santiago, Chile; 9 Millennium Nucleus Center for Plant Systems and Synthetic Biology, Pontificia Universidad Católica de Chile, Santiago, Chile; 10 Department of Mathematical Engineering, Universidad de Chile, Santiago, Chile; 11 Estación Costera de Investigaciones Marinas and Center for Marine Conservation - Las Cruces, Pontificia Universidad Católica de Chile, Santiago, Chile; 12 Center of Applied Ecology and Sustainability, Pontificia Universidad Católica de Chile, Santiago, Chile; 13 Laboratorio Internacional de Cambio Global, LINCGlobal PUC-CSIC, Santiago, Chile

## Abstract

Understanding the factors that modulate bacterial community assembly in natural soils is a longstanding challenge in microbial community ecology. In this work, we compared two microbial co-occurrence networks representing bacterial soil communities from two different sections of a pH, temperature and humidity gradient occurring along a western slope of the Andes in the Atacama Desert. In doing so, a topological graph alignment of co-occurrence networks was used to determine the impact of a shift in environmental variables on OTUs taxonomic composition and their relationships. We observed that a fraction of association patterns identified in the co-occurrence networks are persistent despite large environmental variation. This apparent resilience seems to be due to: (1) a proportion of OTUs that persist across the gradient and maintain similar association patterns within the community and (2) bacterial community ecological rearrangements, where an important fraction of the OTUs come to fill the ecological roles of other OTUs in the other network. Actually, potential functional features suggest a fundamental role of persistent OTUs along the soil gradient involving nitrogen fixation. Our results allow identifying factors that induce changes in microbial assemblage configuration, altering specific bacterial soil functions and interactions within the microbial communities in natural environments.

## Introduction

Soil microbial communities are recognized as being extremely diverse^1,2^ and as the fabric that supports the diverse soil ecosystem functions upon which macroscopic organisms depend^3–6^. Recent studies of microbial communities have greatly benefited from the development of techniques to sequence ribosomal genes without the need for cultivation^7^. These studies provide insights into the importance of environmental factors such as pH^8,9^, temperature^10^ and relative humidity^11,12^ in soil microbial structure and composition.

The extreme conditions faced by microbial communities inhabiting the Atacama Desert soils provide a unique opportunity to test to what extent microbial community structure is resistant to a strong environmental gradient facing multiple natural stressors^13,14^. To understand the impact of the environmental variables on community structure is a necessary step if scientific studies are to provide answers to global scale issues. In this study, we examined the microbial community across an altitudinal transect previously named Talabre-Lejía Transect (TLT)^15^, where direct anthropogenic interference is minimal to nonexistent. Along this transect of few kilometers long, there is a remarkable pH, temperature and humidity gradient, with acidic soils, lower temperatures and higher relative humidity at high elevations and alkaline soils, higher temperatures and lower relative humidity at lower elevations^15^. We studied the compositional structure of the community and constructed two co-occurrence networks representing two sections that divided the TLT gradient. Using network analysis, we examined changes in putative ecological interactions among microbial Operational Taxonomic Units (OTUs) or ‘nodes’, as well as their associations to physicochemical and nutritional variables. Network comparisons based simultaneously on node 16 S rRNA gene sequence identity and topological similarity within the co-occurrence networks allowed us to examine the nature of the ecological rearrangements that take place in the microbial community when facing contrasting environments.

L-GRAAL, the graph alignment method we used here to examine changes in network structure, overcomes general computational needs from previous approaches^16,17^ while allowing for visual representation and interactive examination of important network attributes. To our knowledge, this is the first time the method is applied to microbial systems biology, which by itself represents a significant advance in microbial network comparisons that expands from recent topological characterizations of co-occurrence networks^18^, and provides a comprehensive way to understand topological shifts among members from two networks. We show here that this method provides a glimpse into the nature of the changes in microbial communities that can foster resistance and resilience to contrasting environmental conditions.

## Results

### Talabre-Lejía transect (TLT) exhibits environmental variability

**Figure 1:**
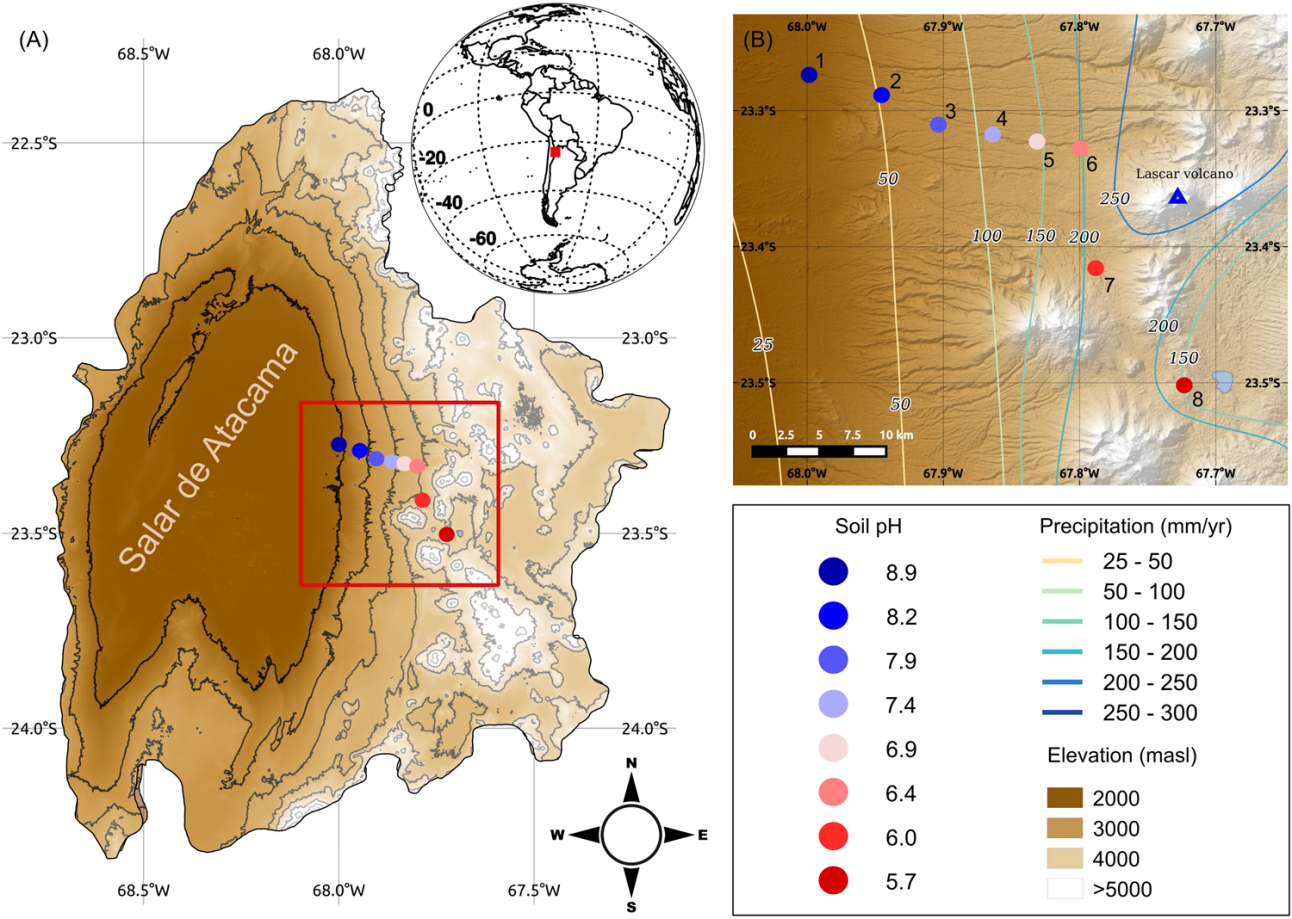
Study and sampling sites. Regional context of the study site in northern Chile showing (**A**) the location of the Salar de Atacama and adjacent Andes and (**B**) an elevation model indicating the sampling sites (colored dots) along Talabre-Lejía Transect (TLT). The software used to create the map was QGIS 2.10 using STRM30^54,55^ elevation model (Data: SIO, NOAA, U.S. Navy, NGA, GEBCO) and Landsat 8 Satellite image (Data available from the U.S. Geological Survey).

Physicochemical and nutritional characteristics of soil differed markedly along the TLT (Supplementary information Table 1). In general, lower elevations showed higher soil temperatures and lower humidity than upper elevations, which was consistent with the rainfall gradient that increases with altitude (Fig. 1; Supplementary information Table 1). Regarding the nutritional variables, they displayed differential trends along the TLT (Supplementary information Fig. 1). At higher elevations, Fe, P, Zn, total C and Cu are enriched, K and Ca exhibit lower concentrations and N derivatives, Mg, S and Na remain constant along the transect. Thus, most of the nutritional variables changed across the altitudinal gradient, indicating contrasting environments in TLT despite the small spatial scale. As shown in a previous report^15^, a strong gradient of soil pH was observed along our eight sampling locations, with values ranging from 8.8 (site 1; lower elevation) to 5.7 (site 8; higher elevation) (Fig. 1; Supplementary information Table 1). Nutrients in the form of total C, P, K and Ca correlated significantly with changes in pH (Spearman’s rho p < 0.05), concordant with previous observations at this site^15^ (Supplementary information Table 1). Total C (0.37–1.1%) and total N contents (0.02–0.06%) were higher than values reported for soils located near our transect^19^, whereas P contents (2.33–26.33 mg/kg) were between one to three orders of magnitude lower at our research site. Micronutrients including Fe, Cu and Zn, negatively and significantly correlated with soil pH (Supplementary information Table 1, Spearman’s rho p < 0.05), which reflects the tightly bound of micronutrients to the soil at high pH^20^.

To examine whether the distribution of sites along the TLT could be explained by the environmental variables (Supplementary information Table 1), we explored their associations in a multivariate space (Supplementary information Fig. 2). Our results showed that the component that mainly explained the ordination of the sites along the transect (PC1, 37.7%), separated sites at low elevation (sites 1, 2, 3 and 4) from those at high elevations (sites 5, 6, 7 and 8). For this reason, for posterior analyses, we divided the TLT transect in two sections: Section 1 (sites 1 to 4) and Section 2 (sites 5 to 8), which were markedly different in pH and nutritional variables.

### TLT microbial diversity is modulated by environmental stressors

A total of 2,798,737 good quality 16 S rRNA gene sequences were obtained. The number of total reads, mapped reads and selected reads, as well as most microbial diversity estimates (Richness, Shannon and Phylogenetic diversity (PD) indices) differed quite markedly among sites (Supplementary information Table 2). Based on the number of observed OTUs, the highest bacterial richness was observed between neutral and slightly acidic sites 6 and 7 (pH 6.2 and 5.9, respectively), at around 4,000 meters above sea level (m a.s.l.) along the TLT (Supplementary information Table 2). These same sites presented the highest microbial diversity values (Shannon and PD indices). In total, 4,437 OTUs were identified along the three replicates of the eight sites when mapped to Greengenes database^21^. As we only selected OTUs that where identified in at least two out of three replicates for posterior analyses, the final number of identified OTUs was 3,072 (Supplementary information Table 2). These OTUs accounted for over 90% of the total microbial relative abundance.

In all the sites analyzed, about 50 to 75% of the relative abundance was accounted by less than 50 OTUs, indicating that few OTUs gave account for most of the bacterial abundance present in Atacama soil microbiomes (Supplementary information Fig. 3).

**Figure 2:**
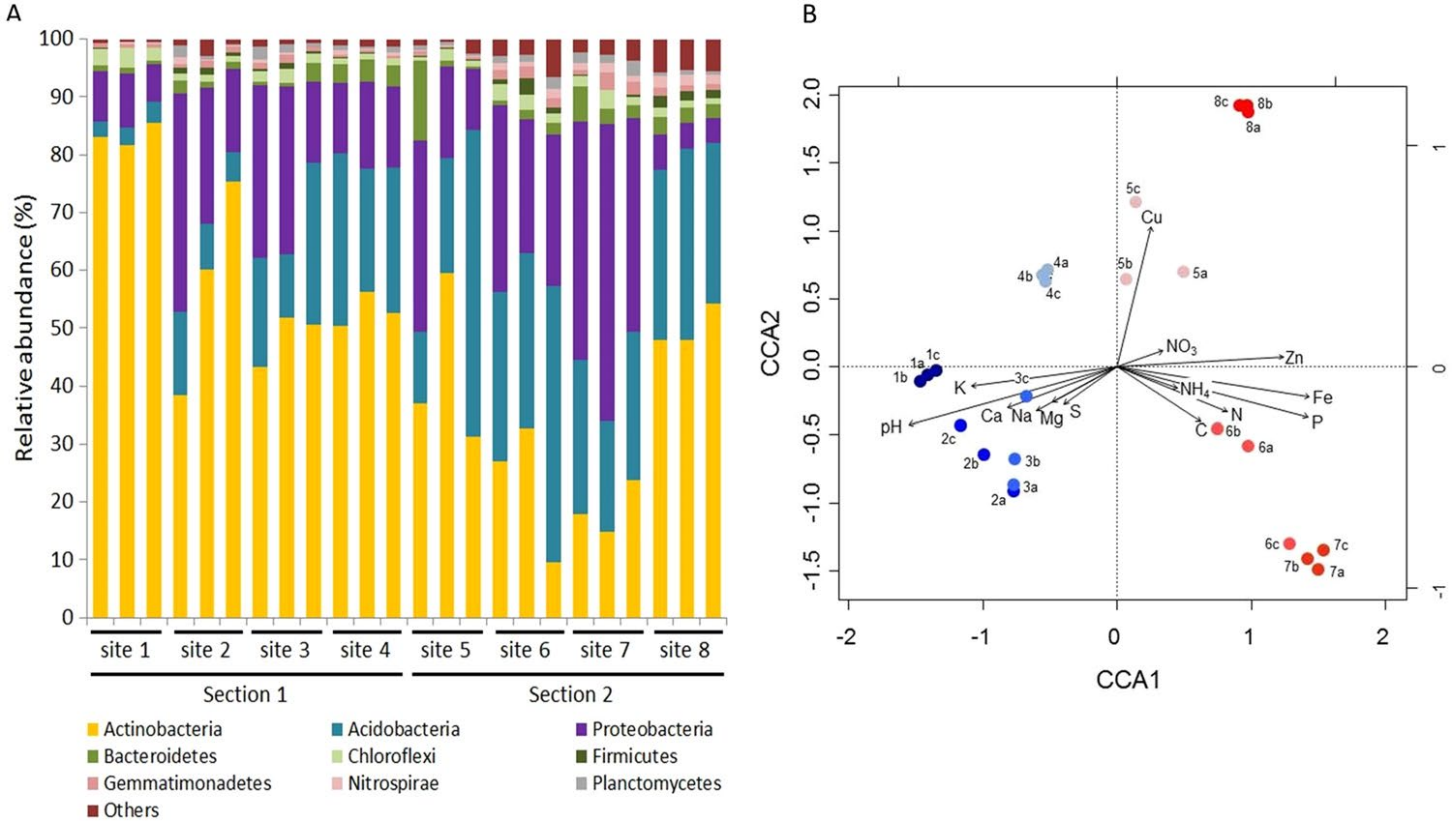
Taxonomic structure of bacterial communities in relation to physicochemical and nutritional variables. (**A**) Triplicates of relative abundances of soil bacterial phyla based on massive sequencing of OTUs in eight sites of TLT. Others: relative abundances <1% in all samples. (**B**) CCA ordination diagram of the bacterial relative abundance data in relation to abiotic variables. Numbers from 1 to 8 represent the sampling sites at  TLT (Fig. 1). Black vectors represent the direction of the variables that could explain sites ordination.

The taxonomic composition of bacterial communities in our soil samples encompassed 24 phyla (Supplementary information Table 3; Fig. 2A). To examine the effect of the pH, temperature and relative humidity gradient on microbial community structure in more detail, we evaluated whether the relative abundance of different phyla changed in a predictable way. The relative abundance of three phyla (Acidobacteria, Gemmatimonadetes and Nitrospirae) changed significantly (Spearman´s rho p < 0.05) with soil pH and temperature, with no indication of unimodal relationship (Supplementary information Table 3; Supplementary information Fig. 4), while relative humidity changed positively and significantly only with the phylum Acidobacteria (Supplementary information Table 3).

Finally, to examine which of the measured variables in TLT were most closely associated to variation of the microbial community (Supplementary information Table 1), we explored their relationship in a multivariate analysis (Fig. 2B). This Canonical Correspondence Analysis (CCA) showed that the concentrations of K and Ca along with pH, were positive and significantly associated (p < 0.01, after Bonferroni correction) to the microbiomes of samples from Section 1 (sites 1 to 4), while C, N (including total N, NO_3_ and NH_4_), P, Fe and Zn were more related (p < 0.01) to microbiomes of samples from Section 2 (sites 5 to 8).

### TLT co-occurrence microbial networks from Section 1 and Section 2 are dissimilar

Inference of microbial ecological interactions was obtained from patterns of occurrence of distinctive 16 S rRNA sequences, physicochemical parameters and soil nutrient content. Co-occurrence networks were constructed using CoNet algorithm, as described previously^22,23^ (Supplementary information Fig. 5).

The network of OTUs from Section 1 (Supplementary Dataset Table 4) contained 484 nodes (476 OTUs and 8 variables) connected by 3,404 edges (2,460 co-presences and 944 exclusions), while for Section 2 (Supplementary Dataset Table 5), the network contained 597 nodes (591 OTUs and 6 variables) and 3,270 edges (2,716 co-presences and 554 exclusions). OTUs that were part of the networks (476 in Section 1 and 591 in Section 2) accounted for 73.87% and 68.39% of the average relative abundance present in Sections 1 and 2, respectively. Moreover, Section 1 and Section 2 networks exhibited 244 and 359 exclusive OTUs (henceforward, noncore OTUs), respectively, and 232 shared OTUs (core network OTUs).

Although some attributes of the networks were preserved between sections, such as the clustering coefficient or the degree to which nodes in a graph tend to cluster as well as the shortest path and path length, other parameters changed noticeably (Supplementary information Table 6). Network density and heterogeneity changed the most; Section 1 network had more average connections and a higher density of hub nodes than Section 2 network. In terms of aggregate attributes, both networks followed an Erdos-Renyi degree distribution, in which most of the nodes are connected randomly (data not shown).

**Figure 3:**
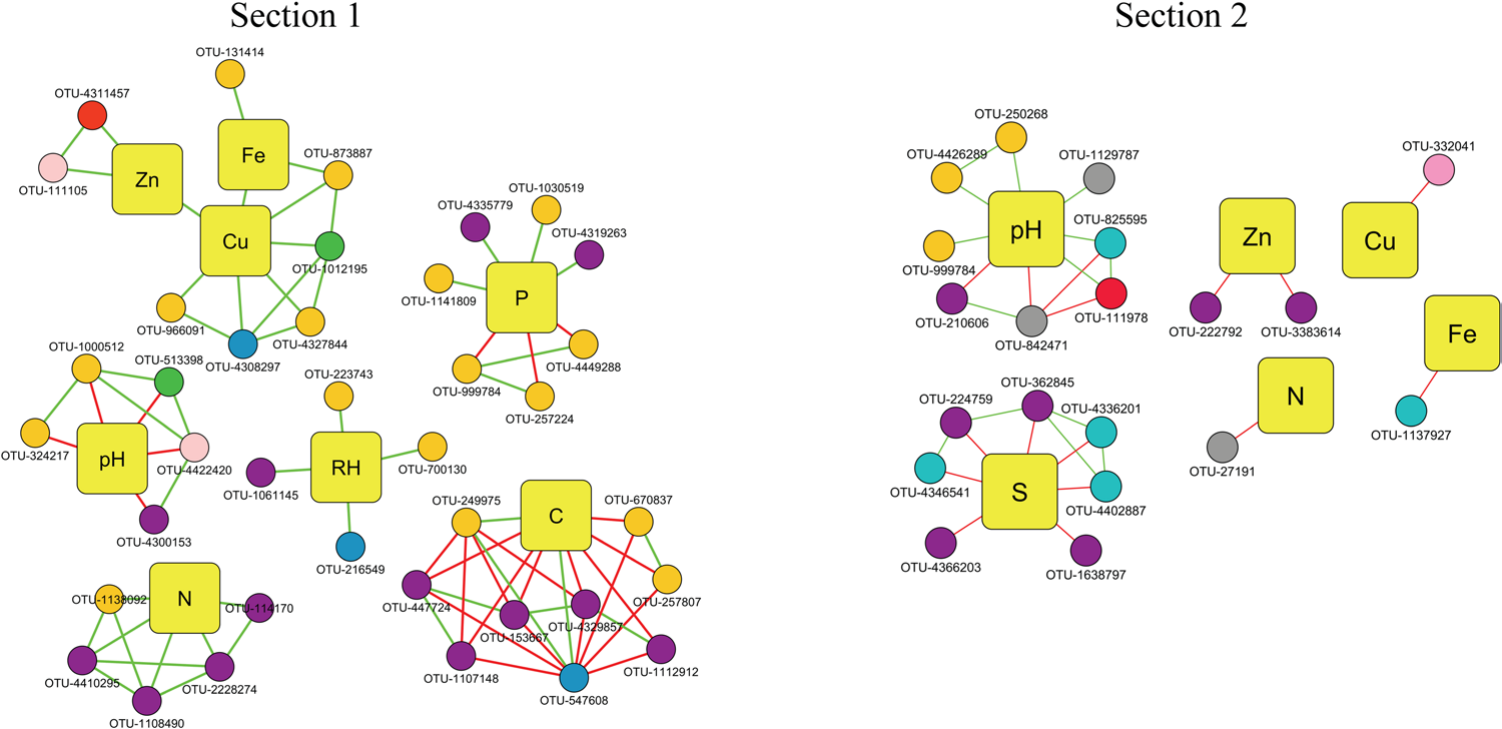
Section 1 and 2 subnetworks. Squared nodes correspond to physicochemical parameters and circle nodes correspond to OTUs. Same color nomenclature for circle nodes and edges from Supplementary information Fig. 5 are used: light blue, Acidobacteria; dark yellow, Actinobacteria; green, Bacteroidetes; light green, Chloroflexi; light yellow, Cyanobacteria; dark green, Firmicutes; pink, Gemmatimonadetes; blue, Nitrospirae; grey, Planctomycetes; purple, Proteobacteria; orange, Verrucomicrobia; red, Armatimonadetes, FBP, TM7 or WPS-2; green edge, positive correlation; red edge, negative correlation.

With respect to the abiotic variables used in networks constructions, the nutrients Zn, Cu, Fe, N, P and C, together with soil pH and relative humidity, were found to be directly connected by 43 edges in the network from Section 1 (28 positive and 15 negative), establishing six subnetworks that are represented in Fig. 3. One of these subnetworks contained Fe, Cu and Zn, which were all directly connected to each other. On the other hand, Zn, Cu, Fe, N, S and soil pH were the variables significantly and directly correlated in the network from Section 2, which added up 20 edges (6 positive and 14 negative). Other six subnetworks were formed in the network from Section 2, each one including only one physicochemical or nutritional variable, thus, no direct connections among abiotic variables were shared in Section 2 network (Fig. 3). The most connected variables were C (9 edges), Cu (7 edges) and P (7 edges) within Section 1 network, while in the network from Section 2, pH (8 edges) and S (7 edges) where the most connected.

**Figure 4:**
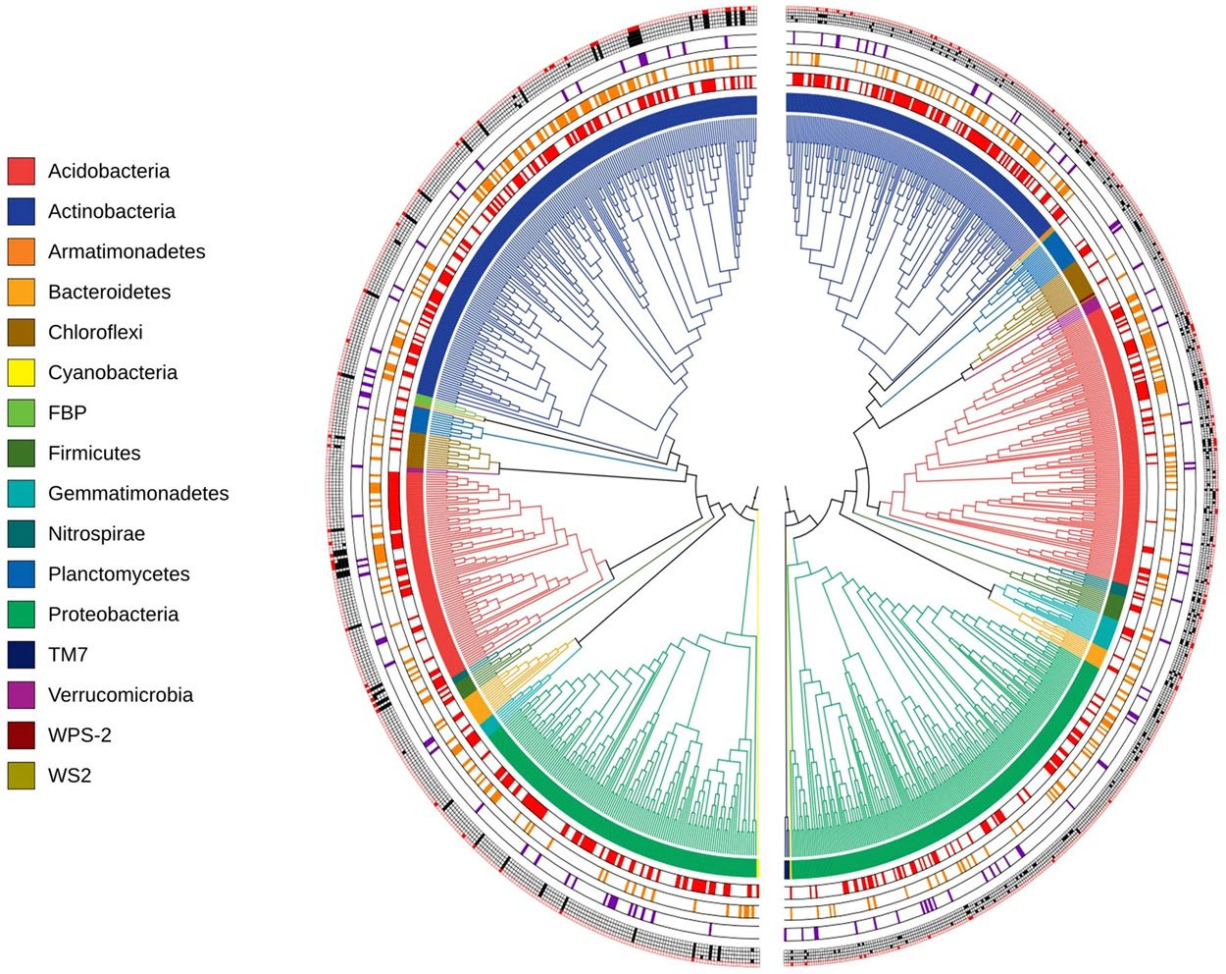
Phylogenetic trees of bacterial networks from Section 1 and 2. (Left) Section 1 network phylogenetic tree; (Right) Section 2 network phylogenetic tree. From the innermost semi-circles: semi-circle (1) and internal tree colors illustrate the taxonomy (phylum) of each OTU; semi-circle (2) shows the type of distribution of every OTU in the network: core (red) or noncore (white); semi-circle (3) illustrates the abundance of every OTU: highly abundant (>0.1% relative abundance in orange) or lowly abundant (<0.1% relative abundance in white); semi-circle (4) shows the centrality value of every OTU: highly connected (>10% higher centrality values in purple) or lowly connected (<10% centrality values in white); semi-circle (5) illustrates correlation values of OTUs relative abundance with abiotic variables (from innermost: Fe, Cu, Zn, N and pH): highly correlated (>10% significant values in black for Fe, Cu, Zn and N; for pH in red) or lowly correlated (<10% significant values in white).

Taxonomic and phylogenetic relationships between the networks were visualized with the Interactive tree of life (iTOL) (Fig. 4). OTUs from Section 1 network were predominantly Actinobacteria (42.2%), with a smaller representation of Proteobacteria (27.7%) (Supplementary Dataset Table 4). In contrast, OTUs from the Section 2 network predominantly belonged to Proteobacteria (34.0%), while Actinobacteria (26.9%) was the second most abundant phyla (Supplementary Dataset Table 5).

Detailed analysis of OTU’s attributes, such as centrality in the network and relative abundance, showed the existence of phylogenetic clusters of OTUs that accounted for some of the differences between the networks (Fig. 4, Supplementary Dataset Table 4; Supplementary Dataset Table 5). For instance, the phylum that had the highest density of core OTUs in the network from Section 1 was represented by Acidobacteria (57.8%), while in Section 2 network, the majority of the core OTUs were Actinobacteria (62.3%). The most abundant OTUs (>0.1% relative abundance) in both networks were generally found to belong to the phyla with the highest number of members, and were mostly core rather than noncore OTUs (Fig. 4; Supplementary Dataset Table 4; Supplementary Dataset Table 5).

In the case of relative abundances of OTUs that significantly and highly correlated (10% higher correlation values) with the physicochemical and nutritional variables common to both networks (pH, Fe, Cu, Zn and N), we noticed that in Section 1 network, 31 of the OTUs that correlated with one of these variables, also correlated with the other 4 variables, a feature that was never observed in Section 2 network (Supplementary Dataset Table 4; Supplementary Dataset Table 5). Moreover, from the 31 OTUs with multiple correlations to variables in the network from Section 1, five also presented high centrality values.

### Nitrogen metabolism highlights in the core bacterial community

We analyzed microbial putative functions displayed by core and noncore OTUs from both networks by Functional Annotation of Prokaryotic Taxa (FAPROTAX) and compared them with the whole bacterial community hypothetical functions present in the transect (Supplementary information Tables 7, 8 and 9). First, we found that the functional roles identified are generally conserved along the entire TLT when considering the most abundant functional categories (>1% relative abundance) displayed by core and noncore OTUs from Sections 1 and 2, such as chemoheterotrophy and nitrate reduction. However, remarkable differences were also observed, highlighting nitrogen fixation, which was significantly higher (p < 0.05, ANNOVA test) in core OTUs (1.8% ± 0.8 relative abundance) compared to noncore OTUs from Sections 1 and 2 (0.07% ± 0.03 and 0.16% ± 0.10 relative abundances, respectively). Also, this putative function was significantly augmented (p < 0.05, T-student test) in core OTUs when compared with the hypothetical functional capacities of the complete community along the transect (0.61% ± 0.2 relative abundance) (Supplementary information Table 7). Other predicted functions associated to nitrogen metabolism were also significantly increased in the core OTUs when compared to the entire TLT, among them aerobic nitrite oxidation and nitrification (Supplementary information Table 7). Conversely, noncore OTUs displayed functional roles in greater abundance (higher than 0.1%) than the complete TLT bacterial community in categories such as fermentation (Section 1; Supplementary information Table 8) and human pathogens and animal parasites or symbionts (Section 2; Supplementary information Table 9).

### Topological graph alignment of co-occurrence networks reveals the impact of environmental variables on OTUs taxonomic composition

L-GRAAL allowed us to align OTUs from Section 1 and Section 2 networks if they shared: (i) same or similar relations within their co-occurrence network (i.e., similar number of theoretical motif participation) and (ii) same or similar OTU sequences (i.e., similar taxonomy). These two methods to align OTUs among Section 1 and Section 2 networks shed light on two different aspects of ecological robustness of microbial communities. First, if the same or closely similar OTUs (in terms of DNA sequence) are aligned together, it means that these nodes establish similar ecological relationships within the microbial network, despite large changes in network structure and environmental stress. Hence, the nodes are themselves ecologically resistant to environmental variability, and the pattern of ecological interactions is preserved, which likely confers robustness to the microbial network. Second, aligned OTUs may be distinctively different in term of sequence (different ‘species’). These OTUs may be found in both networks (core OTUs), but establishing different relationships with other OTUs in their respective environments, and therefore potentially playing different roles. Such a pattern would suggest the existence of some level of ecological redundancy and compensatory relationships within the microbial community as a source of network robustness. Conversely, aligned OTUs may be unique to each network (non-core OTUs), suggesting that different environmental stressors represent constraints leading to the establishment of similar ecological relations^24^ by different microbial entities.

These features used to align OTUs between networks are complementary to each other and shed light into the nature of microbial network re-arrangements when facing different environmental stressors. All combinations of alignments were investigated via the parameter alpha, which reviews the relative importance of OTUs relations and OTUs sequence identities among networks (Supplementary information Fig. 6, panel A). For instance, alpha equals 1 indicates that the OTU alignment considers sequence similarity only (Supplementary information Fig. 6, panel C). Reversely, for alpha equals 0 (Supplementary information Fig. 6, panel B), OTUs were aligned only using their respective topological similarity based on graph-let decomposition. Thus, two OTUs from Section 1 and Section 2 were aligned if they possessed a similar topological feature. The extensive analysis of all alignments for alpha values over the range between zero and one to decipher the best consensus between topological and taxonomic features, edge correctness (EC) and symmetric substructure (SS) scores (Supplementary information Fig. 6, panel A) indicated that the alpha that represented the best consensus to perform an alignment analysis was 0.6.

Also, for the sake of method validation, Section 1 and Section 2 networks were compared to networks generated randomly by edges permutation, such that, homologous OTUs were aligned emphasizing solely a topological change, mimicking a randomization of samples. Section 1 and Section 2 networks were closer (i.e., higher scores) than these same networks compared with random ones (Supplementary information Fig. 7). Considering this, our results indicated that both networks are pertinent to define the configuration of bacterial community networks in both sections of TLT. In addition, Alpha equals to 0.6 remained the best consensus to align section networks (EC = 25.18%, SS = 17.25%).

**Figure 5:**
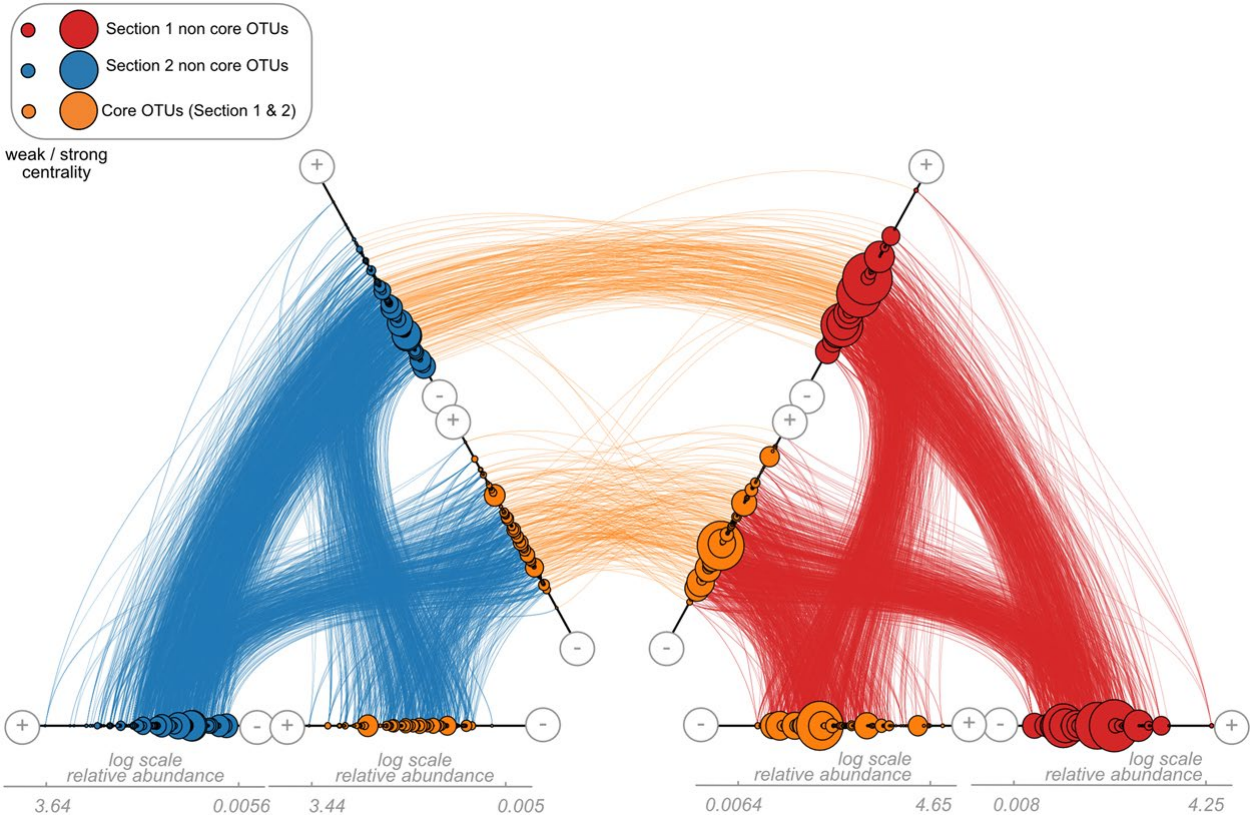
Hiveplot graphic alignment of Section 1 and Section 2 networks at alpha 0.6. Nodes of the networks are ranked by their relative abundance (in log-scale) and duplicated on two axes to represent the co-occurrence network structure (respectively, blue and red for Section 1 and Section 2). On each axis, nodes were split into two sub-axes, where external sub-axes depict noncore OTUs (present only in a single network), whereas the central sub-axes show OTUs in both sections (i.e., 232 core OTUs). The size of the nodes shows the centrality value of each OTU within their respective network, based on the centrality score. The central edges between two hiveplot graphs (orange) represent the alignments between nodes according to the alpha value of interest. Hiveplot alpha 0.6 online visualization at https://pydio-bird.univ-nantes.fr/pydio_public/a79507.

Our graph alignment analysis showed that Section 1 network was more self-connected than Section 2, as indicated by the higher centrality scores (larger size of nodes) of core and noncore OTUs (Fig. 5, orange and red nodes). The hiveplot representations of the communities showed that most abundant OTUs tend to be less connected (Fig. 5), suggesting again the existence of low abundant keystone OTUs in the networks. A large fraction (159 out of 232; 68.5%) of the core OTUs that were aligned to themselves between networks, also retained the pattern of association within the networks. When we evaluated the potential functional roles of the 159 OTUs that aligned to themselves in both networks (Supplementary information Table 7), we found that the relative abundances of potential functional categories in core and aligned core OTUs was practically the same, with nitrogen fixation as one of the most abundant (>1% of relative abundance) hypothetical functions in both sets of OTUs.

Regarding noncore OTUs from Section 1, they displayed 28 functional categories, from which 24 were shared with their aligned OTUs from Section 2 (Supplementary information Table 8), whereas Section 2 noncore OTUs shared 25 out of 28 functional categories with their aligned OTUs (Supplementary information Table 9). The functions that were exclusively assigned to Section 1 noncore OTUs (methanol oxidation, dissimilatory arsenate reduction, arsenate detoxification and plastic degradation) and the functions exclusive from their aligned OTUs in Section 2 (dark oxidation of sulfur compounds, hydrocarbon degradation, methanotrophy and anoxygenic photoautotrophy H2 oxidizing) added less than 0.3% of the total relative abundance in each section. Similarly, the three exclusive potential functions of noncore OTUs from Section 2 (dark oxidation of sulfur compounds, hydrocarbon degradation and methanotrophy) and the three functions exclusive for their aligned OTUs in Section 1 (dissimilatory arsenate reduction, methanol oxidation and plastic degradation) added 0.08% and 0.24% of the total abundance in each section (Supplementary information Table 9). Thus, in terms of functional composition, noncore OTUs and their aligned OTUs displayed essentially the same global functional capacities.

## Discussion

In this work, we assessed whether changes in diversity and community structure take place among the microbial communities inhabiting a 56 km long altitudinal transect across the central Atacama Desert. This transect, named as TLT, exhibits a pronounced pH, temperature and relative humidity gradient and major differences in the contents of several micro and macro-nutrients that allowed us to examine spatial differences in the bacterial community in a natural, small spatial scale and environmentally contrasting gradient transect.

By applying CCA, we detected that taxonomic changes observed across the TLT were also driven by a combination of pH and nutritional factors, which is consistent with the observation that changes of pH values among soil samples showed significant correlations with many nutritional variables (including micronutrients Fe, Cu and Zn) (Supplementary information Table 1). Interestingly, the clustering of the sites in Sections 1 and 2 along the TLT became more evident after the CCA than after the PCA, which only considers environmental variables (Supplementary information Fig. 2), highlighting the tight connection between the microbial community and the environment, and suggesting that the microbial communities contribute to define and differentiate the sites along the TLT. These analyses indicate that the differential pH along the gradient is causing the effects in nutrient availability, and therefore, it is the main factor controlling the bacterial community structure in TLT. Variation in soil pH has been associated with changes in bacterial composition at high taxonomic levels^8^, while bacterial diversity in edaphic environments has been unimodally correlated to pH, reaching a peak at near-neutral conditions and decreasing towards more acidic and more alkaline conditions^8^. However, most of our knowledge about the ecological effects of pH on microbial communities comes from studies carried out in artificial pH gradients^25^ or in highly managed urban systems (with fertilizers and compost applications, among others)^26^, which reduce the complexity and variability of environmental conditions to which these organisms are subjected to in the natural environment. Although important insights on mechanistic relationships can be gained under controlled conditions, it is difficult to assess to what extent a diverse bacterial community is resistant or resilient to naturally variable conditions. At the other extreme, soil studies have documented differences in microbial communities found under naturally contrasting pH conditions, but located in different regions of the world^1,8^, making it difficult to separate historic and phylogeographic effects from local ecological responses.

In this scenario, we took advantage of a sharp pH gradient found across a comparatively small spatial scale in the Atacama Desert to assess the effect of *in situ* pH on microbial composition and community structure. We constructed two co-occurrence networks representing the two sections of the TLT that grouped sites with high or low pH: Section 1, which corresponded to alkaline sites; and Section 2, which corresponded to acidic sites. Using network analysis, we examined changes in putative ecological interactions among microbial OTUs or ‘nodes’, as well as their association to physicochemical and nutritional variables. Even though co-ocurrence microbial networks from Section 1 and Section 2 showed differences in some of the parameters measured including connectivity and network attributes, they both followed an Erdos-Renyi degree distribution^27,28^. Although this is not common in most biological networks (including bacterial OTUs), most of which follow a classical power-law distribution, such a degree distribution has also been observed in other microorganism communities. Co-occurrence networks of Archaea extracted from soil samples exhibited an Erdos-Renyi degree distribution^27^, which according to the authors suggests a nearly neutral co-occurrence. This means that the interaction between species from acid and alkaline networks are equally likely^29^.

In both networks, the most connected OTUs (10% higher centrality values), which are often proposed to be critical or keystone components for network stability^30^, belonged to the same three phyla (Actinobacteria, Acidobacteria and Proteobacteria). Interestingly, these hub nodes are not dominant in abundance, indicating that while central and abundant nodes belong to the same general taxa, they are not the same species. This implies that highly abundant and core OTUs that belong to Actinobacteria, Acidobacteria and Proteobacteria may not be as critical for network dynamic and stability as some of the low abundant but highly connected ‘species’, which therefore may play a keystone role in network dynamical properties^31,32^.

The putative functional roles identified along the entire TLT when considering the most abundant functional categories were largely conserved. However, some differences were observed when analyzing core and noncore OTUs. Core OTUs appeared to be highly and significantly abundant in hypothetical functions associated to nitrogen metabolism, especially nitrogen fixation (Supplementary information Table 7). This suggests that core OTUs play a role in the maintenance of bacterial community structure in face of environmental change due to their capacity to easily adapt or resist to variations in physicochemical and nutritional factors by making available one of the most important elements for microbial survival: nitrogen. Conversely, and as expected, noncore OTUs displayed hypothetical functional roles in greater abundance than the complete TLT bacterial community (higher than 0.1% of relative abundance) in categories that are not fundamental in the preservation of the community along the transect.

The analysis of network alignment, which is based simultaneously on node 16 S rRNA gene sequence identity and co-occurrence network topological similarity, allowed us to examine the nature of the ecological rearrangements that take place in the microbial community when facing contrasting environments. In particular, we examined whether association patterns between community members were preserved between the networks. We found that a large fraction of core OTUs persisted at both extremes of the TLT gradient and also established similar patterns of association with other community members. If such association patterns among members play a significant role on network dynamics and stability^24,33^ and reflect functional properties within the community, as shown for protein networks^34^, then this set of ‘resistant’ OTUs may confer some level of robustness or resilience to the entire community when facing a radically altered environment. In support of this conjecture, core OTUs and their respective aligned OTUs in the other network displayed essentially the same global potential functional capacities, and more importantly, nitrogen fixation was one of the most abundant functions in both networks. Given that most of these core OTUs aligned to themselves do not have high centrality values and the majority are distributed among the three most abundant phyla (Actinobacteria, 40.3%; Proteobacteria, 32.7% and Acidobacteria 14.5%), the fundamental and maintained putative role along the transect of nitrogen fixators would have not been identified by other more traditional community or network analyses.

On the other hand, when network alignment analysis was performed among noncore OTUs of different taxonomies, but with similar network topologies, substantial rearrangements were observed in the microbial community, in such a way that Section 1 noncore OTUs seemed to fill ecological roles previously occupied by Section 2 noncore OTUs. However, in terms of potential functional composition, noncore OTUs and their aligned OTUs displayed essentially the same global functional capacities. Hence, patterns of interaction appear to play a role in community hypothetical functions and, therefore, dynamics, as shown in many theoretical studies^34^. Together, these results reveal fair levels of redundancy in the bacterial community as well as ecological constraints that lead to the preservation of some key network attributes despite changes in the players.

In this study we observed that despite the large physicochemical (mainly pH) and nutritional differences that influence the bacterial community structure along the TLT, both network association patterns are quite persistent. Some of this apparent resilience between TLT sites seems to be due to the roughly 20% of OTUs that persist across the gradient and maintain similar interaction patterns within the community. Thus, our results slightly modify the traditional idea of community resilience by proposing that relationships between OTUs may also be resilient. We showed that these core OTUs with similar interaction patterns also maintain ecological roles in the community (i.e. nitrogen fixation), some of which can be crucial for microbial survival. The robustness of the bacterial community seems also to be associated to ecological rearrangements, in such a way that an important fraction of the OTUs come to fill the interactive roles occupied by other OTUs in the network when facing strong environmental changes. In summary, our study allows a glimpse into the type of ecological and functional changes that may modulate resilience and adaptation of microbial communities to changing environments.

## Methods

### Background and site description

The study site corresponds to an altitudinal gradient along the western slope of the Andes Mountain in the Atacama Desert, Chile (Fig. 1). This 56 km gradient, named in previous studies Talabre-Lejía Transect (TLT), spans c. 2000 m of elevation, from approximately 2,500 up to 4,500 m a.s.l. Mean annual precipitation and mean annual temperature vary across the gradient from an extremely low ~10 to ~200 mm yr^-1^ and ~13 to ~4 °C, respectively^35^ (Fig. 1).

### Sample collection

Three replicated soil samples (~100 g each) were collected in April 2013 (austral summer) under sterile conditions from the upper 5 to 10 cm of soil surface at eight distinctive locations (24 samples in total) within TLT (Fig. 1). Each replicate of soil was homogenized *in situ* before storage. From each replicate, a subsample of 50 g was immediately stored on dry ice for microbial analyses (approximately one week of storage), while another 50 g subsample was air-dried, sieved (≤2 mm) and stored for physicochemical analyses. Additional samples were collected in years 2012, 2013 and 2014 at these same sites to provide a characterization of physicochemical and nutritional temporal variability of the sites.

### Environmental and soil physicochemical measurements

Soil temperature and relative humidity were measured in the field with portable iButton DS1923 Hygrochron sensors located between 5 and 10 cm soil depth. Soil pH was determined in a 1:1 w/w soil deionized water ratio^36^ and electrical conductivity in a 1:5 w/w soil deionized water ratio^37^. Nutrients analyses were carried out by the Laboratorio Agroanálisis UC, Facultad de Agronomía e Ingeniería Forestal, Pontificia Universidad Católica de Chile, according to the methods established by the Normalization and Accreditation Commission (CNA) of the Chilean Society of Soil Science^38^. The nutrients that were measured were N Total, NO_3_, NH_4_, C Total, P, S, K, Ca, Na, Mg, Fe, Cu and Zn.

### Soil DNA extraction and sequencing

DNA was extracted from subsamples collected from each of the three soil samples at each site using the Qiagen kit DNeasy Blood & Tissue combining manufacturer’s instructions and the CTAB based method^39,40^. Five grams of soil were resuspended in 5 ml extraction buffer [100 mM Tris-HCl; pH 8, 100 mM Na EDTA; pH 8, 100 mM Na_2_HPO_4_, 1.5 M NaCl, 1% (w/v) CTAB] and then 10 mg/ml (final concentration) of Lyzosyme was added and mixed by vortex, followed by incubation at 37 °C for 1 h with constant mixing. The mixture was centrifuged at 2000 × g for 5 min at room temperature and the supernatant fluid was transferred to a clean tube, to which 3 ul of Pronase (100 mg/ml) were added and then incubated at 37 °C for 1 h with gentle and constant mixing. Then, 1 ml of 20% (w/v) SDS was added and incubation continued at 65 °C for another 1 h, gently shaking the tubes. The mixture was then centrifuged at 6000 × g for 10 min at room temperature and the supernatant fluid was transferred to a new clean tube and mixed with vortex with 1 ml of Qiagen AL Binding Buffer. The mixture was incubated at 65 °C for 10 minutes with gentle shaking, and then 1 mL of Ethanol 100% was added and the mixture transferred into DNeasy mini spin column to continue the kit protocol. The integrity of the DNA was evaluated by electrophoresis in the Agilent 2200 TapeSation equipment and then stored at −20 °C until DNA analyses.

Microbial DNA was amplified using a bacteria-specific primer set, 28 F (5′-GA GTT TGA TCM TGG CTC AG-3′) and 519 R (5′-GWA TTA CCG CGG CKG CTG-3′), flanking variable regions V1-V3 of the 16 S rRNA gene^41^ with barcode on the forward primer. Amplification was performed using the Qiagen Kit HotStarTaq Plus Master Mix under the following conditions: initial denaturation at 94 °C for 3 min followed by 28 cycles, each set at 94 °C for 30 seconds, 53 °C for 40 seconds and 72 °C for 1 min, with a final elongation step at 72 °C for 5 min. After amplification, PCR products were checked in a 2% agarose gel to determine the success of the amplification and the relative intensity of the bands. At this point, the three replicate samples for microbial analyses were pooled together in equal proportions based on their molecular weight and DNA concentrations. Pooled samples were purified using calibrated Agencourt Ampure AMPure XP beads. These processed PCR products were used to prepare DNA libraries following Illumina TruSeq DNA library preparation protocol. Sequencings were performed at the Molecular Research DNA laboratory (Shallowater, TX, USA) on an Illumina MiSeq platform in an overlapping 2 × 300 bp configuration with a minimum throughput of 20,000 reads by sample.

### Processing of Illumina sequence data

16 S rRNA raw amplicon sequences were processed and analyzed following previously described protocols^42,43^. Briefly, sequences were joined (overlapping pairs) and grouped by samples following the barcodes and then barcodes were removed. Then, sequences <150 bp or with ambiguous base calls were removed. Remaining sequences were filtered using the USEARCH clustering algorithm at 4% sequence divergence to remove chimeras and clusters consisting of only one sequence (i.e. singletons)^44,45^. Finally, sequences were quality filtered with Mothur v.1.22.2^46^ with the minimal quality average set to 30. All sequence data used in this study have been deposited in the Sequence Read Archive (SRA) of the National Center for Biotechnology Information (NCBI) under the BioProject accession number PRJNA358231.

### Sequence analysis and taxonomic identification using Greengenes database

Sequences were analyzed with the software Quantitative Insights Into Microbial Ecology (QIIME v1.8.0)^47^. Briefly, we used QIIME script ‘pick_closed_reference_otus.py’ to extract all 16 S rRNA reads from the amplicon data that matched GreenGenes r16S database^21^, release gg_otus_13_08) at 97% of similarity or 3% divergence, with the taxonomy of the resulting Operational Txonomic Units (OTUs) assigned directly from the closest sequence match (“mapped reads”). The OTU picking process was performed with USearch v6.1.544^44,45^ using QIIME default parameter values (-s 0.97 –z True–max_accepts 1–max_rejects 8–word_length 8 –minlen 64–usearch61_sort_method abundance). This closed-reference phylotype picking process generated 4,437 OTUs comprising 623,530 reads across all 24 samples. OTUs unassigned or assigned to mitochondria and chloroplast were removed. Singletons were also removed. For analyses, we selected reads that mapped with OTUs that where identified in at least two out of three replicates (“selected reads”), in order to analyze data using representative OTUs from each site so we could make robust comparative analyses between the different bacterial communities. Using this criterion, 3,072 OTUs were included in the analyses.

### Microbial diversity and composition and Multivariate analyses

To characterize microbial diversity patterns, we calculated alpha OTU diversity by randomly subsampling (without replacement) each soil sample using the alpha_rarefaction.py script in QIIME. The Shannon and Faith’s Phylogenetic Diversity (PD) indices, along with the observed number of OTUs (‘richness’), were calculated. Rarefaction curves for each of these metrics were obtained by serial subsampling in increments of 389 sequences and 10 iterations per increment, to a standardized 3,900 sequences per sample. This number represented the second lowest number of curated sequences obtained across our samples. The samples with the lowest number of sequences were excluded from this analysis.

To establish the association among sites in terms of the 14 correlated abiotic variables (physicochemical and nutritional), we conducted a Principal Component Analysis (PCA) using the prcomp function of the R package stats version 3.2.3 using centered and scaled variables (pH, N total, NO3, NH4, C total, P, S, K, Ca, Na, Mg, Fe, Cu and Zn).

To examine compositional changes in the microbial community across the gradient and to establish associations with soil environmental and nutrient variables, OTU abundance data was converted into incidence data (presence/absence) and then we conducted a Canonical Correspondence Analysis (CCA) using R package vegan v2.3–2^48^. Our data consisted of 3,072 OTUs by 8 sample matrix and the same 14 variables used for PCA.

### Co-occurrence microbial networks

As a first approximation to examine changes in the ecological structure of the microbial network between contrasting pH levels and other environmental conditions, we contrasted the microbial networks found in the extremes of the pH gradient, i.e. neutral to alkaline soils (sites 1 to 4, here called Section 1 network) and neutral to acidic soils (sites 5 to 8, Section 2 network, Fig. 1). To examine the structural association between OTU’s and soil physicochemical and nutritional parameters, we included the pH, Relative Humidity, Temperature, N total, C total, P, S, Cu, Fe, Zn, as nodes in the networks. We selected these physicochemical and nutritional parameters for the network display because they better explained the association of OTUs in the eight sites sampled of TLT in the CCA and/or are important factors for bacterial growth^49–51^. OTUs that occurred in less than five samples were discarded.

Links or edges of the microbial networks were obtained from OTU occurrence data. Significant co-presences or exclusions across the samples were identified by the CoNet method^23^ using a multiple ensemble correlation (guessing pair = 50, row_minocc filter = 10). Four similarity measures were calculated: Bray Curtis and Kullback-Leibler non-parametric dissimilarity indices; Pearson and Spearman rank correlations. A distribution of all pairwise scores between phyla was computed for each site to enrich the network with phyla nodes. Based on OTU’s and phyla’s distributions, initial thresholds were selected such that the initial network contained 1,000 positive and 1,000 negative edges consistent across all four correlation measures. For each measure and each edge, 1,000 renormalized permutation and bootstrap scores were generated according to^22^. Co-occurrence network model was displayed by Cytoscape^52^, which revealed the statistics of the networks including the Number of nodes, Clustering coefficient, Shortest paths, Path length, Density and Heterogeneity.

For the sake of sensibility analysis, networks were randomized. Given a section network, we built a graph called random network, which contained similar nodes and similar number of edges. The building process consisted in shuffling edges, such that one randomly considered edge between two corresponding nodes (called herein n_i and n_j) became an edge between two nodes randomly chosen among the list of nodes, but not n_i and n_j. This random procedure was performed a number of times equivalent to the number of total edges. This procedure was conducted via in house python script and using networkx and random library.

### Topological graph alignment of co-occurrence networks

To examine the extent to which the individual OTU’s change the way they interact with other OTUs in the network when inhabiting contrasting pH environments, co-occurrence Section 1 and Section 2 networks were compared via the graph-alignment technique L-GRAAL^34^. Originally applied to compare protein-protein interaction networks, this method aligns nodes of two graphs when they share both similar topological properties (i.e., for each OTU in each network, the graph-let decomposition depicts the number of theoretical motifs in which the given OTU is involved) in their respective graph and similar labeling properties (i.e., sequence).

In the graph alignment analyses, nodes of Section 1 and Section 2 networks are ranked by their relative abundance (in log-scale) and duplicated on two axes to represent the co-occurrence network structure (Blue and Red for Section 1 and Section 2, respectively, Fig. 5). On each axis, nodes were split into two sub-axes, where external sub-axes depict noncore OTUs (present only in a single network), whereas the central sub-axis show OTUs in both pH conditions (i.e., 232 core OTUs). The size of the nodes shows the centrality value of each OTU within their respective network, based on the degree centrality score. The central edges between two hiveplot graphs (orange) represent the alignments between nodes (i.e., OTUs) according to the alpha value of interest.

### Functional structure of TLT

To shed light into the functional structural rearrangement undergone by the microbial network across the pH gradient, we estimated the potential functional roles of OTUs using the Functional Annotation of Prokaryotic Taxa (FAPROTAX), following the method of Louca and coworkers (2016)^53^. Briefly, each taxonomically annotated OTU was compared against each FAPROTAX annotation rule in an automated way. The hypothetical functional roles were calculated as averages from sites 1 to 8 for core and core aligned to themselves OTUs, and from sites 1 to 4 for Section 1 OTUs, and sites 5 to 8 for Section 2 OTUs.

## Electronic supplementary material

Supplementary information

Supplementary Dataset Table 4

Supplementary Dataset Table 5
