# Supplementary material for: Structure and co-occurrence patterns in microbial communities under acute environmental stress reveal ecological factors fostering resilience
Source: Sci Rep. 2018 Apr 12;8:5875. doi: 10.1038/s41598-018-23931-0 (PMC5897386; doi:10.1038/s41598-018-23931-0)
Supplement: Supplementary file 1 — Supplementary information [file 41598_2018_23931_MOESM1_ESM.docx]

**Supplementary information**

**Structure and co-occurrence patterns in microbial communities under acute environmental stress reveal ecological factors fostering resilience**

Dinka Mandakovic^1,2^, Claudia Rojas^3^, Jonathan Maldonado^1,2^, Mauricio Latorre^1,2,3,4^, Dante Travisany^4^, Erwan Delage^5^, Audrey Bihouée^6^, Géraldine Jean^5^, Francisca P. Díaz^2,7,8,9^, Beatriz Fernández-Gómez^1,2,4^, Pablo Cabrera^1,2^, Alexis Gaete^1,2^, Claudio Latorre^2,7,8,13^, Rodrigo A. Gutiérrez^2,9^, Alejandro Maass^2,4,10^, Verónica Cambiazo^1,2^, Sergio A. Navarrete^11,12,13^, Damien Eveillard^5^, Mauricio González^1,2^.

^1^Bioinformática y Expresión Génica, Instituto de Nutrición y Tecnología de los Alimentos, Universidad de Chile, Santiago, Chile.

^2^Center for Genome Regulation (Fondap 15090007), Universidad de Chile, Santiago, Chile.

^3^Instituto de Ciencias Agronómicas, Universidad de O'Higgins, Rancagua, Chile.

^4^Mathomics, Center for Mathematical Modeling, Universidad de Chile, Santiago, Chile.

^5^LS2N, UMR CNRS 6004, IMT Atlantique, ECN, Université de Nantes, Nantes, France.

^6^l'institut du thorax, INSERM, CNRS, Université de Nantes, Nantes, France.

^7^Departamento de Ecología, Pontificia Universidad Católica de Chile, Santiago, Chile.

^8^Institute of Ecology and Biodiversity (IEB), Santiago, Chile.

^9^Millennium Nucleus Center for Plant Systems and Synthetic Biology, Pontificia Universidad Católica de Chile, Santiago, Chile.

^10^Department of Mathematical Engineering, Universidad de Chile, Santiago, Chile.

^11^Estación Costera de Investigaciones Marinas and Center for Marine Conservation - Las Cruces, Pontificia Universidad Católica de Chile, Santiago, Chile.

^12^Center of Applied Ecology and Sustainability, Pontificia Universidad Católica de Chile, Santiago, Chile.

^13^Laboratorio Internacional de Cambio Global, LINCGlobal PUC-CSIC.

Correspondence and requests for materials should be addressed to M.G. (E-mail: mgonzale@inta.uchile.cl)


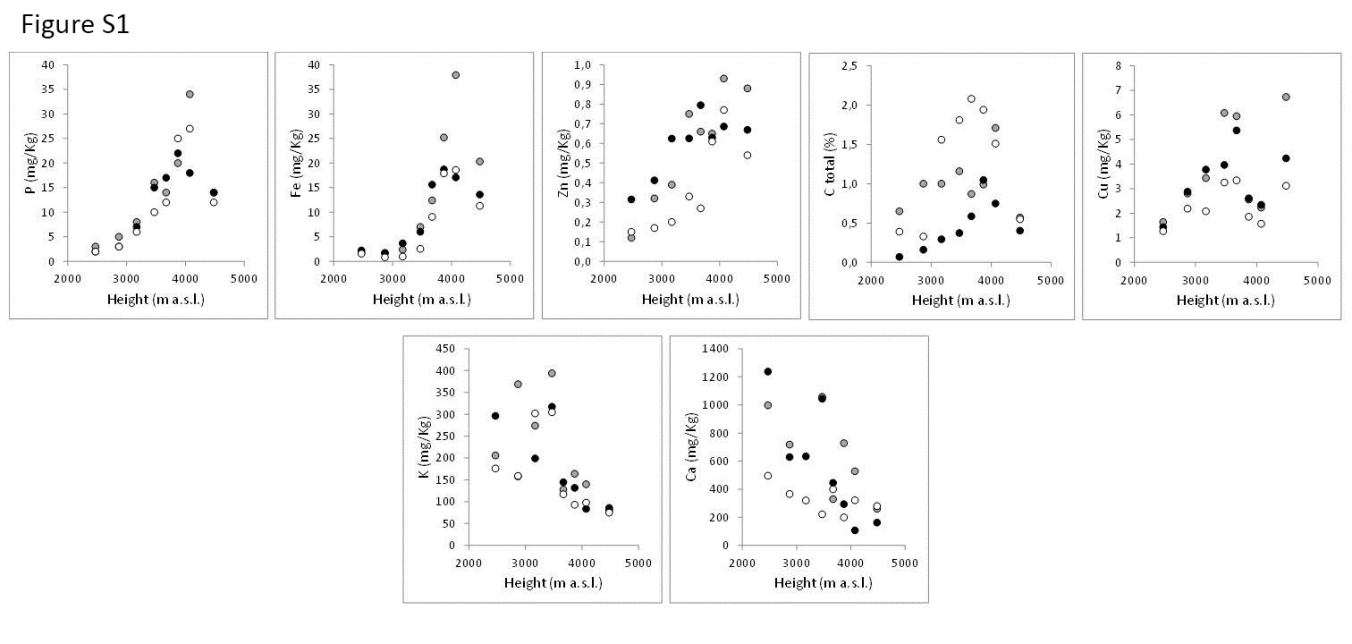


**Supplementary information Fig. 1. Nutritional variables along the transect.** Grey circles represent samples from 2012; black circles represent samples from 2013 and white circles represent samples from 2014.

**
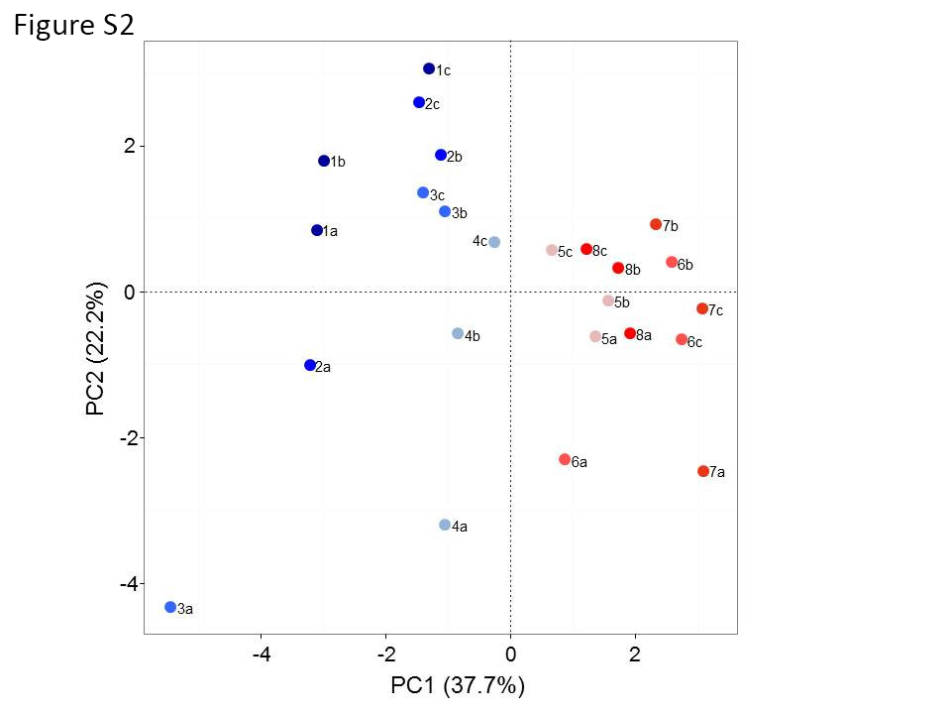
**

**Supplementary information Fig. 2. Principal Component Analysis (PCA) ordination diagram of the sites along the TLT.** Numbers from 1 to 8 represent the sampling sites in TLT (Figure 1).


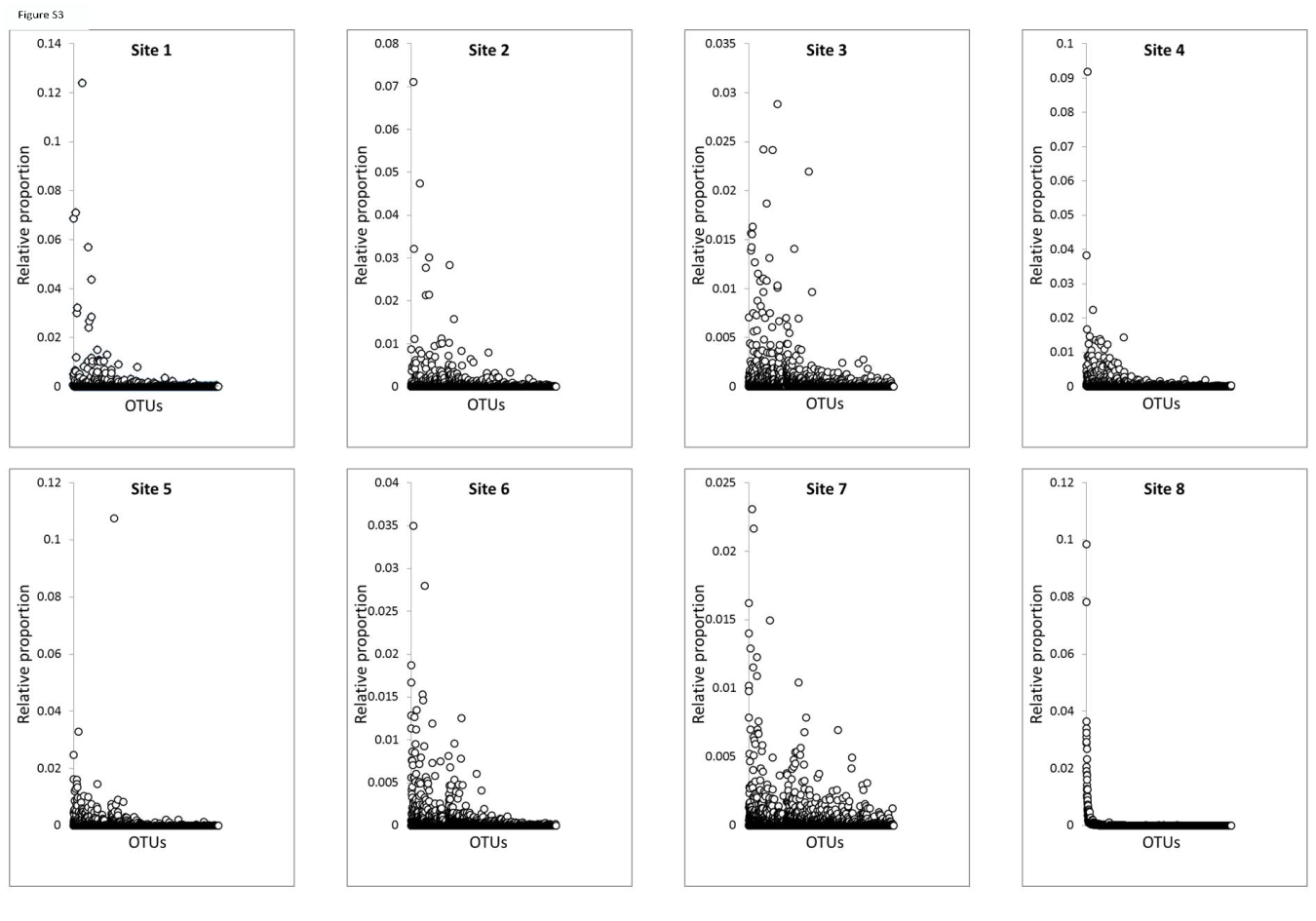


**Supplementary information Fig. 3.** **Distribution of the relative proportion of the OTUs in the 8 sites.**


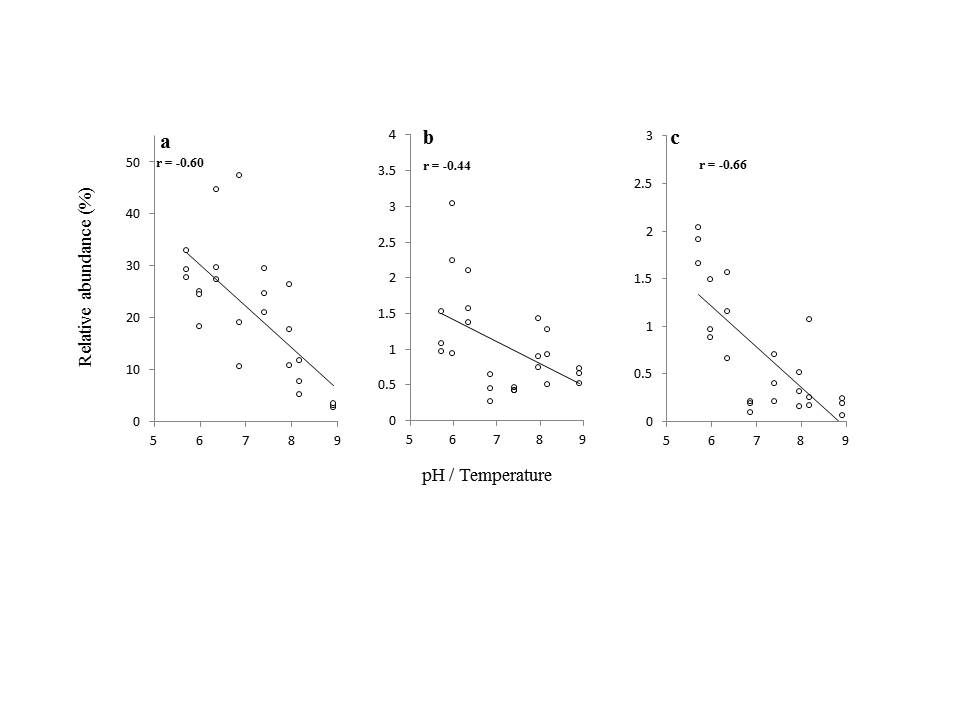
**Supplementary information Fig. 4.** **Relative abundance of phyla that significantly correlate with pH and temperature**. Phyla with abundance correlations with pH and temperature with Spearman’s rho p < 0.05 in eight sites from TLT with abundances > 1 % in at least one sample. Triplicates are displayed. (a) Acidobacteria (b) Gemmatimonadetes and (c) Nitrospirae.

**
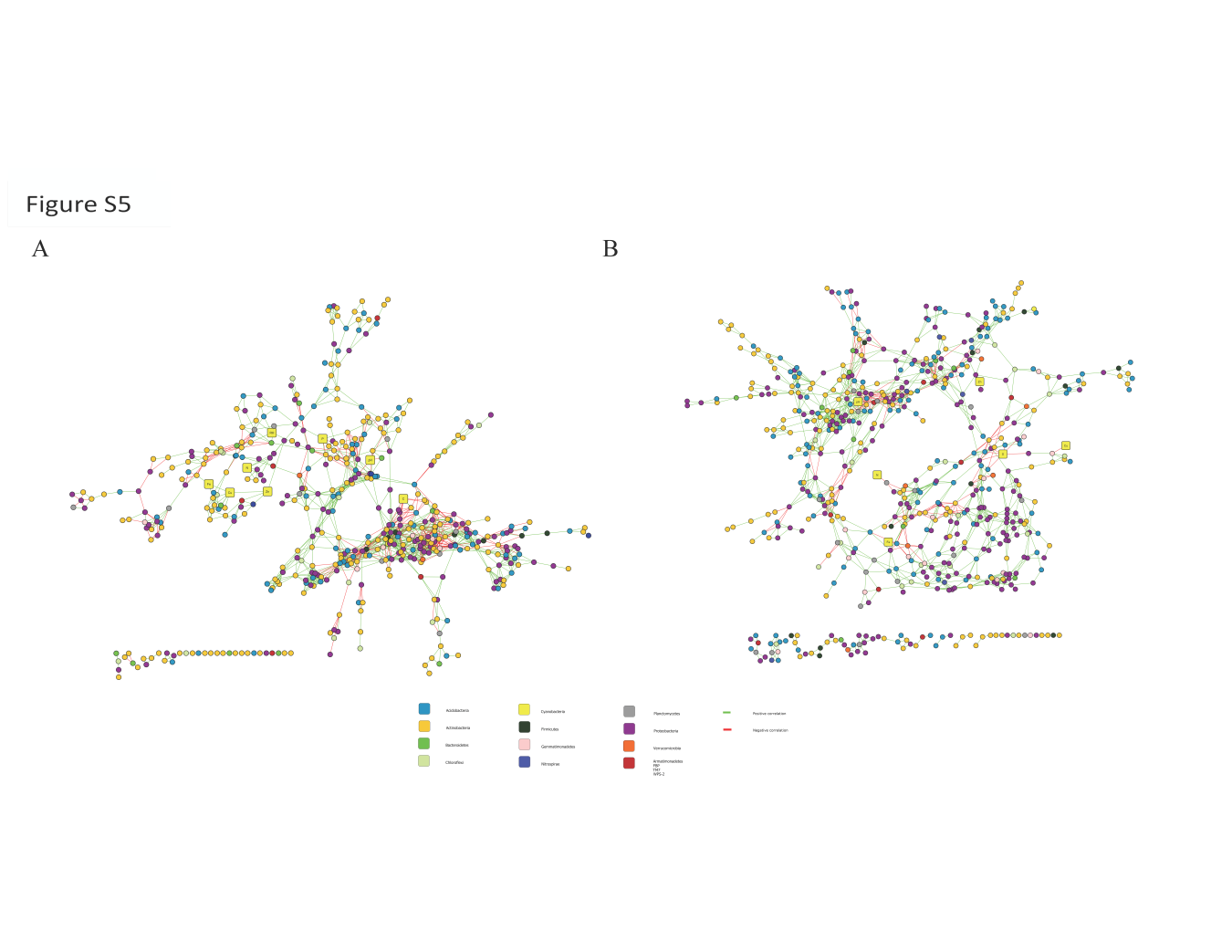
Supplementary information Fig. 5. Section 1 and 2 OTUs co-occurrence microbial networks.** A. Section 1 network. B. Section 2 network.

**
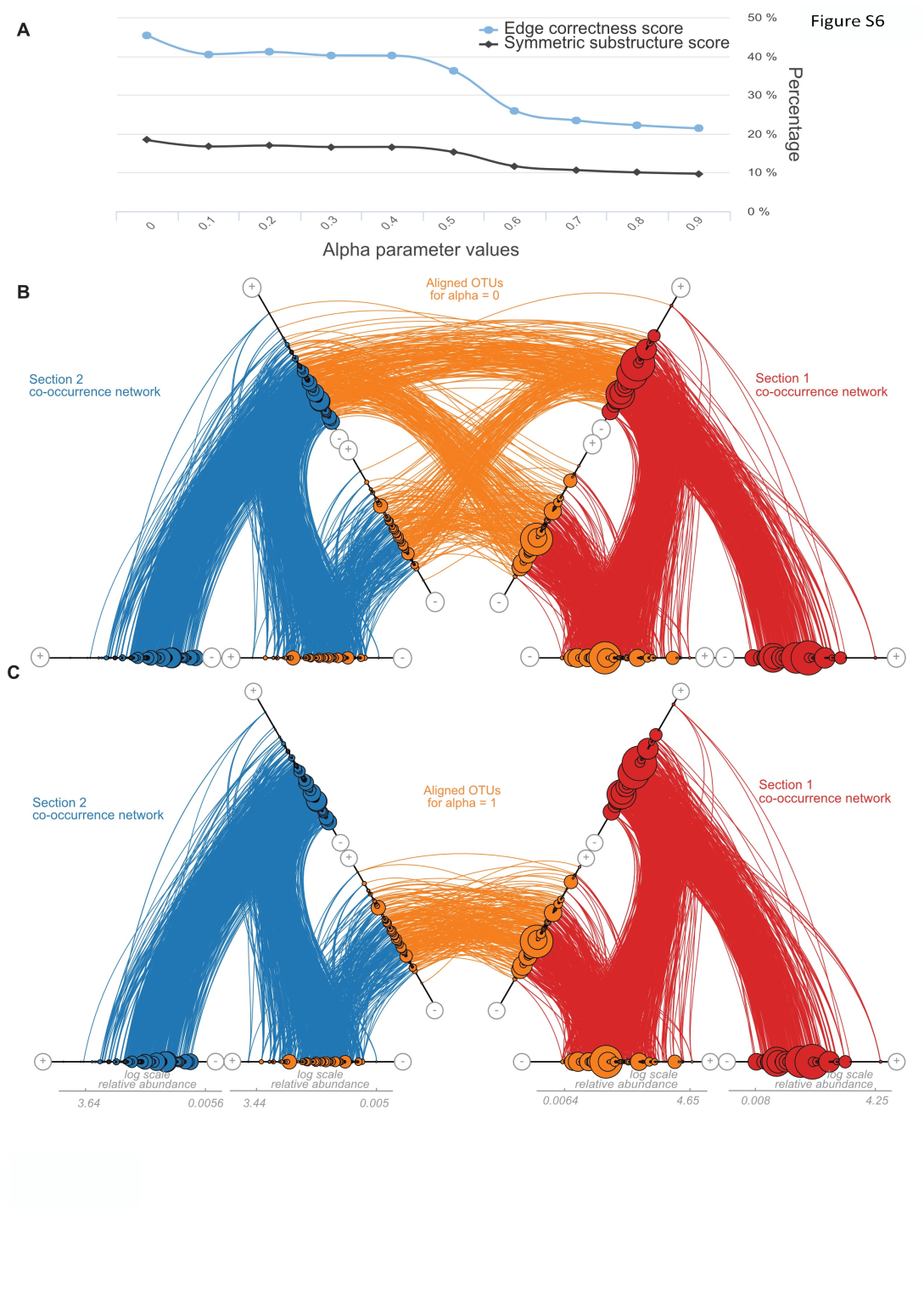
**

**Supplementary information Fig. 6. Alignment of Section 1 and Section 2 graphs for different alpha parameter values.** A. Evolution of topological alignment metrics for different alpha parameter values. Alpha parameter controls the relative importance between topological similarity (alpha = 0) and sequence similarity (alpha = 1). B. Graph alignment for alpha equals to 0. Graph alignment results are summarized as a hiveplot graph were nodes of Section 1 and Section 2 networks are ranked by their relative abundance (in log-scale) and duplicated on two axes to represent the co-occurrence network structure (respectively red and blue for Section 1 and Section 2 networks). Size of nodes is proportional to centrality. Following the Figure 5 nomenclature, nodes in orange are present in both networks (i.e.core), whereas blue and red are specific to each co-occurrence network. Aligned nodes respect similar topological motifs only. C. Graph alignment for alpha equals to 1 following a similar procedure. Aligned nodes respect similar sequences. Hiveplots for alphas 0.0 and 1.0 online visualizations at https://pydio-bird.univ-nantes.fr/pydio_public/a79507.

**
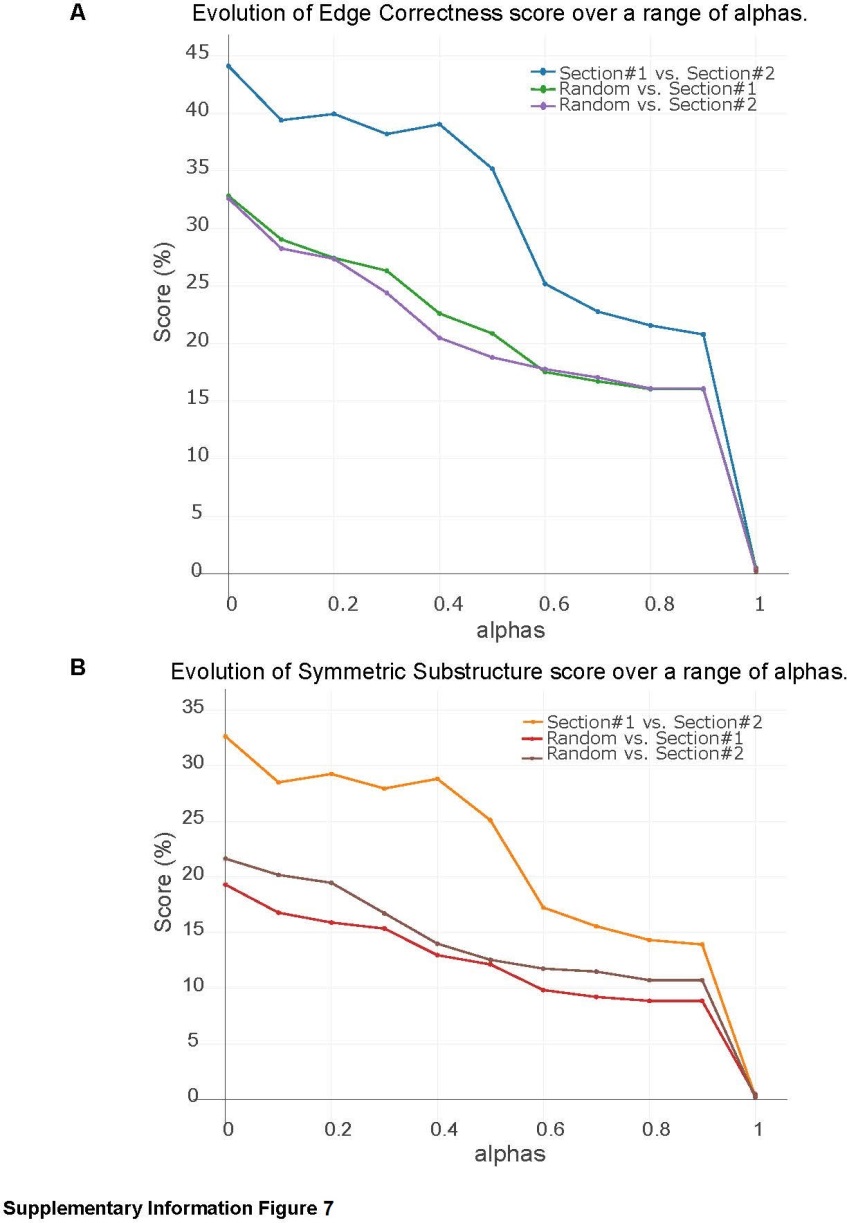
**

**Supplementary information Fig. 7. Comparison of Section 1 and Section 2 networks with generated random networks.** The comparison is performed by two complementary scores: A. edge correctness (EC) and B. symmetric substructure (SS) scores for all alpha values (i.e., ratio of topological constraints vs. sequence homology constraints to align nodes). For the particular alignment of alpha equals to 0.6 (selected for all analyses, see Supplementary Information Figure 7), random networks are more distant from Section 1 (EC = 17.52%, SS = 11.76%) or Section 2 (EC = 17.77%, SS = 9.84%).

**Supplementary information Table 1. Physicochemical and nutritional parameters from sites 1 to 8.**

**Supplementary information Table 2. Alpha diversity.** Microbial diversity indicated by Richness, Shannon diversity and Faith’s phylogenetic diversity (PD). Average values and standard errors (in parenthesis) are shown. Calculations of richness and diversity estimators was based on OTU tables rarefied to the same depth of 3,900 sequences.

**Supplementary information Table 3. Average relative abundance of each phylum in the eight sites and their correlation coefficients with pH, temperature and relative humidity.**

**Supplementary information Table 6. Main network measures.**

**Supplementary information Table 7. Functional categories of core and core aligned to themselves OTUs from Sections 1 and 2.**

**Supplementary information Table 8. Functional categories of Section 1 noncore OTUs and their aligned OTUs in Section 2.**

**Supplementary information Table 9. Functional categories of Section 2 noncore OTUs and their aligned OTUs in Section 1.**
